# Complete genome of the mutualistic symbiont “*Candidatus* Nardonella sp.” Pin-AIST from the black hard weevil *Pachyrhynchus infernalis*

**DOI:** 10.1128/mra.01083-24

**Published:** 2025-02-25

**Authors:** Masaki Mizutani, Minoru Moriyama, Takema Fukatsu, Shigeyuki Kakizawa

**Affiliations:** 1 Bioproduction Research Institute, National Institute of Advanced Industrial Science and Technology (AIST), Tsukuba, Japan; 2 Department of Biological Sciences, Graduate School of Science, University of Tokyo, Tokyo, Japan; 3 Graduate School of Life and Environmental Sciences, University of Tsukuba 13121 , Tsukuba, Japan; University of Maryland School of Medicine, Baltimore, Maryland, USA

**Keywords:** insect endosymbiont, genome, PCR-amplified fragment

## Abstract

The complete genome, 226,287 bps in size, of “*Candidatus* Nardonella sp.” Pin-AIST, an obligatory bacterial endosymbiont of the black hard weevil *Pachyrhynchus infernalis*, was sequenced. The extremely reduced endosymbiont genome is specialized for tyrosine synthesis, which contributes to the hardness of the beetle’s exoskeleton.

## ANNOUNCEMENT

Beetles constitute a highly successful insect group in the terrestrial ecosystem (1). One of the reasons for this success is attributable to their highly sclerotized exoskeleton (2). A clade of bacterial endosymbionts, designated as “*Candidatus* Nardonella spp.” (3, 4), has been identified as contributing to the hard exoskeleton of weevils (5). “*Ca*. Nardonella” has an extremely reduced genome, about 0.2 Mb in size, which is streamlined for synthesis of a single amino acid tyrosine that is needed for the formation and sclerotization of the hard exoskeleton of the host weevils (5).

Here, we sequenced the genome of “*Ca*. Nardonella sp.” Pin-AIST from the Japanese black hard weevil *Pachyrhynchus infernalis* strain AIST, which was collected at Ishigaki Island, Okinawa, Japan, in 2018. This weevil strain has been maintained in our laboratory under 25°C and 16:8 hour light–dark conditions, with adults fed mainly on carrots and larvae fed sweet potatoes (5). The genomic DNA was extracted from dissected larval bacteriomes by using conventional proteinase K digestion and phenol extraction (6) and subjected to long-PCR. The entire 226 kbp genome sequence of *Nardonella* (GenBank: AP018160) was divided into 16 fragments and amplified using 16 primer sets (Table 1). Each primer was 25 or 35 nucleotides long, and the resulting PCR products ranged from 10 kbp to 25 kbp in size. Long-PCR was performed using KOD-One polymerase (TOYOBO) according to the manufacturer’s instructions. All 16 fragments were successfully amplified and purified using the PCR Purification Kit (QIAGEN). For library construction, the purified PCR products were fragmented and tagged using MGIEasy FS DNA Library Prep Set (MGI Tech), circulated using MGIEasy Circularization Kit (MGI Tech), and rolling-amplified using the DNBSEQ-G400RS High-throughput Sequencing Kit (MGI Tech). The constructed DNA Nanoballs (DNBs) were sequenced using the DNBSEQ-G400 (MGI Tech) platform in the 200-bp paired-end mode. The obtained 7,803,814 paired-end reads were mapped onto the reference genome (AP018160) to eliminate host-derived reads, and then the 7,704,067 mapped reads were applied to *de novo* assembly using CLC Genomics Workbench 23.0.4 (QIAGEN). Default parameters were used for all software, unless otherwise specified. The obtained contigs were manually assembled into a single circular genome with 6,811-fold coverage. The final genome was polished by read mapping using CLC Genomics Workbench, and annotation was performed using DFAST pipeline v1.6.0 (7).

**TABLE 1:** PCR primer pairs used in this study

Region	Amplified length (bp)	Forward primer name	Forward primer sequence (5'−3')	Reverse primer name	Reverse primer sequence (5'−3')
1	12,300	1 F	ATTATAATCAAATTATTTAACCAATAAAATTAATA	1 R	TTTTTTAAATCATATGGAGTTAATTCTACAATAAC
2	12,400	2 F	ATGTATTATATATTATAAATAATATTTATTATTAT	2 R	TTCTAAATTTAAAAAATATTAAATACCAAATATCT
3	12,563	3-F2	TATTATTTATCTTATTTAAATTCAG	3 R	TAAAGGGTGAGTAGGTGGTTTTATAAGTCAAATGT
4	12,600	4 F	ACGACAGCCATGCAGCACCTGTCTCAAAGCTTCTT	4 R	ATTTAAATTAAAATTAATTATATAATTATTTATAT
5 + 6	24,800	5 F	TCAAATTTAATTAAAATAAAGTCTGAAATAAATTG	6 R	TTTATCTTTCCATGTTTCAGATGTAGCTAAACTTA
7	12,900	7 F	AGATCCATTTATTAATAAATTAGGAATTCTTGATG	7 R	TTTAACAGGAGTTTCAACTATAATTTTACATAATA
8	12,300	8 F	CCTGATCATAATCCTAATAATAGTTCATCTGAAGA	8 R	TCTGGTTTATTTTTAGAATTAATAAATGATTCTAT
9	12,400	9 F	AGAAAAAGTATTATATTTTGAATCTTATATAGTTA	9 R	ACTTGCATTATAGAAGAATAATTCATTATATCTTT
10	12,500	10 F	TTTTATGTCAAAAAAGATTAAAATTAATGATGATG	10 R	ATTTATAATACTGTAATTAATAAAAGAGATATATC
11 + 12	24,700	11 F	TAGATATATATTCTCTAGTATATTCTACATTTTTT	12 R	AATAAATATAGTTTATATAAAATTTAATGTATATT
13	12,800	13 F	TATTTTTCTTTAATAAACTCCCAATTTTTTAAAAT	13 R	AAATAAAAATATTAATATTAAAAAAAAATTTTATT
14	12,900	14 F	TTTTATAAACTATTTGATAGTATATTAAATATAGA	14 R	TAATGCTTTACATTTATTTAAAATATAAAAAAAAA
15 + 16	24,400	15 F	CAATGAGGTAAAAATTGTTAGTTAATAAAGATTTA	16 R	ATATTAATATATAATCTAATAAACTATTATTTTTA
17	12,500	17 F	TAAAAAGTTTAAAAAAAAATTTATTATTTATAATT	17 R	TATATTAGGATGAATATTTTTTTTCATATATAATT
18	12,600	18 F	TTAAAAATGATTTATCTATATCTACTACAACTAGT	18 R	TTTATTATTTATATTAGATAATATATATTTAACAT
19	10,299	19 F	TATTAAATATAGAAAAAAATGGAAAAATGAAATTA	19 R	ATAATTATTATTTAATTATGTAATTAATTATAAAA

The complete circular genome of “*Ca*. Nardonella sp.” Pin-AIST was 226,287 bp in size with 17.4% G + C content. It encoded putative 212 protein-coding sequences (CDS), 32 transfer RNAs, and two copies of 16S/23S/5S ribosomal RNA operon. The streamlined endosymbiont genome lacked most genes for amino acid synthesis pathways, except for tyrosine synthesis pathway genes. In comparison with the genome of “*Ca*. Nardonella sp.” Pin (AP018160), which was previously determined for *P. infernalis* field-collected at Ishigaki Island in 2012 (5), the genome of “*Ca*. Nardonella sp.” Pin-AIST was almost identical but for 30 nucleotide substitutions and five indels (Table 2 at https://doi.org/10.6084/m9.figshare.28209578.v1). Our newly obtained genome sequence data will contribute to comparative analyses of symbiont genomics and provide insights into the evolution and diversification of complex insect–microbe mutualisms.

## Data Availability

The sequence data have been deposited to DDBJ under the accession numbers AP036057 for assembly and DRR591237 for raw reads. The results of sequence comparison between the determined genome and the reference genome are given in Table 2 at https://doi.org/10.6084/m9.figshare.28209578.v1.
